# Toll-like receptors: versatile players in tumor

**DOI:** 10.3389/fimmu.2026.1735188

**Published:** 2026-06-05

**Authors:** Yang Yang, Ruyu Pi

**Affiliations:** 1 Department of Gynecology and Obstetrics, Key Laboratory of Birth Defects and Related Diseases of Women and Children, Ministry of Education, West China Second Hospital, Sichuan University, Chengdu, China; 2 Shanghai Mental Health Center, Shanghai Jiao Tong University School of Medicine, Shanghai, China

**Keywords:** immune response, regeneration, toll like receptor, treatment resistance, tumor microenvironment, tumor recurrence

## Abstract

Toll-like receptors (TLRs) are a class of pattern-recognition receptors first identified for their role in detecting microbial pathogens and initiating innate immune responses. Beyond pathogen defense, TLRs serve as crucial mediators connecting innate and adaptive immunity and contribute to processes such as tissue repair and remodeling. Owing to their functional versatility, TLRs are involved in multiple stages of tumor progression, presenting both anti- and pro-tumor effect. Here we review the functions of TLRs in physiologic activities, as well as their roles in tumor biology including tumorigenesis, tumor progression, tumor microenvironment, treatment sensitivity, tumor recurrence and patient prognosis. TLRs function as a context-dependent interface where host homeostasis and tumor subversion compete; the additive, synergistic, or antagonistic integration of their signals ultimately determines whether the balance tips toward tumor suppression or progression.

## 1 Introduction

Toll-like receptors (TLRs) are type I transmembrane glycoproteins with evolutionarily conserved structures, existing in a wide variety of species (1, 2). Toll receptor protein was first found in Drosophila’s innate immune responses against fungal infection in 1996 (3, 4). Since then 13 functionally active homologs of Toll receptor--TLRs have been identified in humans and mice, of which TLR1–9 are conserved and functional in both species (5–7). While TLR10 is functional in human, it is non-functional in mice due to a retrovirus insertion (6).

**Figure 1:**
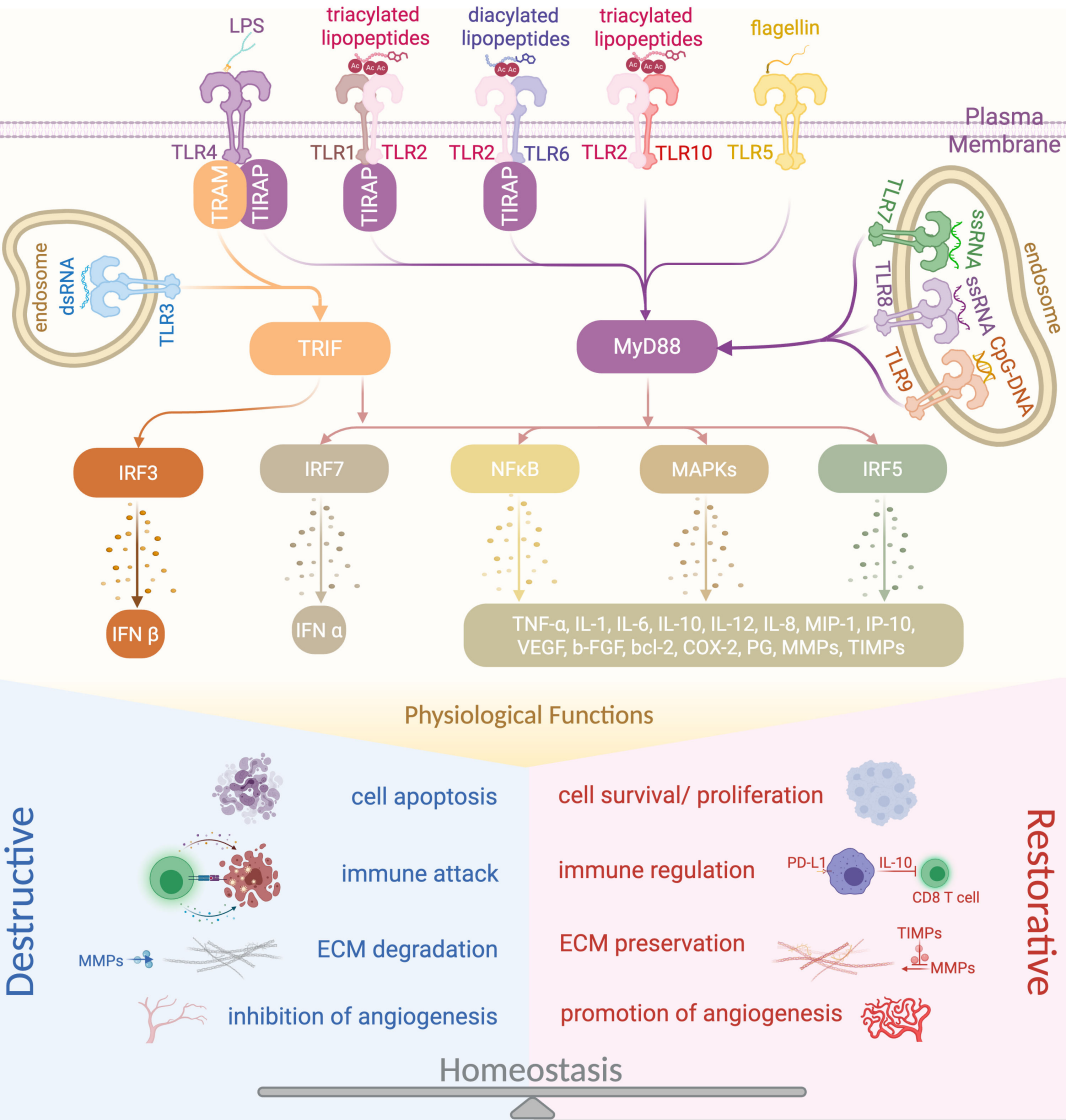
TLR signaling pathways and physiological functions Under physiological conditions, TLRs are located either on the cell surface (TLR1, 2, 4, 5, 6, 10) or within endosomal compartments (TLR3, 7, 8, 9). When engaged by their ligands, TLR dimers initiate signaling through either the MyD88- or TRIF-dependent pathways. Specifically, TLR4 uniquely triggers both the MyD88 (with adaptor TIRAP) and TRIF (with adaptor TRAM) cascades, while TLR3 signals exclusively via TRIF. In contrast, TLR1/2, TLR2/6, TLR2/10, TLR5, and TLR7–9 utilize only the MyD88 pathway, independent of additional adaptor proteins. These signaling events ultimately lead to the induction of type I interferons through IRF3 and IRF7 activation, and to the production of pro-inflammatory cytokines via NF-κB, MAPK, and IRF5 pathways, thereby orchestrating diverse physiological responses and underpinning the multifaceted roles of TLRs in tumor biology. Those functions encompass both the destructive activities against exogenous pathogens and the affected cells and tissues and the restorative activities to the damaged cells and tissues; functions from both sides work in balance to keep homeostasis. (This figure is created with BioRender.com).

TLRs have a broad expression in various tissues, especially in immune active locations, such as the spleen, peripheral blood, the lung and the gastrointestinal tract, with the latter two constantly contact with microbes (8). TLRs also express in vascular system, neural system, skin, reproductive and urinary tract (9–11). In human, before activation, respective TLRs primarily locate in either the cell surface or the intracellular endosomes (4, 12–14) (**Figure 1**), and may travel to different compartments inside the cell after stimulation (15).

TLRs tend to form into dimers after activation by their ligands (12), and subsequently transduce the signals via the myeloid differentiation factor 88 (MyD88) pathway or the TIR domain-containing adaptor protein-inducing interferon-β (TRIF) pathway (16). By the two pathways, a range of downstream molecules can be activated, including interferon (IFN) regulatory factor 3 (IRF3), IRF5, IRF7, nuclear factor κB (NF-κB), mitogen-activated protein kinases (MAPKs) (including ERK, JNK, p38) (16–19). Consequently, type-IFN (IFNα, IFNβ), inflammatory cytokines (TNF-α, interleukin-1 (IL-1), IL-6, IL-10, IL-12, etc.), and chemokines (IL-8, macrophage inflammatory protein (MIP)-1, IP-10, etc.) are produced and released into tissue (20–24). (**Figure 1**).

The ligands activating TLRs can be divided into 3 categories: exogenous, endogenous and synthesized agents. Representative ligands for each TLR are shown in **Table 1**. TLRs can recognize diverse microbial pathogens by their conserved molecular patterns, namely, pathogen-associated molecular patterns (PAMPs) as exogenous ligands, and subsequently initiate immune responses (21, 46). For the endogenous ligands, TLRs also recognize conserved damage-associated molecular patterns (DAMPs) (35), produced by cell death and extracellular matrix (ECM) degradation from tissue damage after trauma or infection, functioning as “danger signals”. The DAMP-TLR ligation is crucial for the initiation of tissue repair (5, 25, 47). TLRs are also key participants in numerous biological processes, such as adaptive immune regulation (48), cellular differentiation and development (49, 50), tissue regeneration (51–53), control of the cell cycle (54, 55), and metabolic regulation (56, 57). (**Figure 1**).

**Table 1:** TLR ligands.

TLR	Exogenous	Endogenous	Synthesized	References
TLR2				(25–34,276)
TLR1/2	triacylated lipopeptides (G-bacteria)	HSPs, HMGB1, biglycan, hyaluronan, β-defensin, versican, amyloids, CEP	Pam3CSK4 (triacylated lipopeptide)	
TLR2/6	diacylated lipopeptides (mycoplasma, G+bacteria), zymosan (fungus)			
TLR2/10	triacylated lipopeptides and other PAMPs shared by TLR1			
TLR3	dsRNA (virus)	self dsRNA, mRNA	polyIC(polyinosinic‐polycytidylic acid)	(25, 35–37)
TLR4	LPS (G- bacteria), mannanes (fungus), glycoinositol phospholipids (trypanosoma)	HMGB1, biglycan, hyaluronan, β-defensin, fibrinogen, heparin sulfate, angiotensin II	lipid A mimetics	(25, 27, 38)
TLR5	flagellin (bacteria)	HSPs, HMGB1		(35, 39, 40)
TLR7	ssRNA (viral), siRNA(bacteria)	self ssRNA, self DNA	imiquimod (imidazoquinoline derivatives)	(25, 35, 41)
TLR8	ssRNA (viral)	self ssRNA	Motolimod (imidazoquinoline derivatives)	(25, 35, 42)
TLR9	CpG DNA (bacteria and virus)	self DNA, self RNA, HMGB1	CpG ODNs	(25, 35, 43–45)

CEP, ω-(2-carboxyethyl) pyrrole (lipid oxication end product); CpG ODNs, CpG oligdeoxyneocleotides; dsRNA, double stranded RNA; HMGB1, high-mobility group box protein 1; LPS, lipopolysaccharide; ssRNA, single stranded RNA.

TLRs demonstrate diverse functions during tumor initiation, progression, and the reshaping of the tumor microenvironment (TME), where their activities may become enhanced or dysregulated, leading to either tumor-suppressive or tumor-promoting outcomes (58–60). Harnessing the anti-tumor potential of TLRs has yielded promising advances in cancer immunotherapy, and several synthetic TLR agonists have already received FDA approval for clinical application (61). Overview of both pre-clinical and clinical studies targeting TLR in anti-tumor therapies can be found in our previous review (62). However, many questions still remain, including potential immunosuppression induced by TLR agonists (63, 64).

Here we briefly summary the physiological functions of TLRs. By compiling decades of published articles and clinical trials, we have summarized the multifaceted roles of TLR in different specific aspects of tumor development, relapse, and treatment. Finally, we discussed factors that may influence the TLR function in tumors, which are important to be bear in mind when interpreting implications from pre-clinical studies as well as clinical trials and when designing future studies; on top of the above, we discussed the research prospect.

## 2 Physiological functions of TLRs

TLRs are involved in many physiological activities to maintain homeostasis, including inflammation, immune responses, tissue regeneration, and remodeling (65) (**Figure 1**). Considering the impact on tissues, we categorize TLR activities into “destruction” (immune attack to both pathogens and infected host cells) and “restoration” (tissue repair after damage to restore the original structure).

### 2.1 Destruction: anti-infectious immune response

The TLRs were first reported to against microbe infection in innate immunity. However, characteristics of TLR-mediated anti-infection responses also present in other immune activation situations such as vaccination, auto-immune diseases, and tumor development. Here we present TLR functions in antimicrobial immunity as a typical example to understand the immune stimulation potential of TLRs.

In the host defense process, after recognizing pathogen components, TLRs subsequently initiate an immediate antimicrobe innate immune response and develop a long-lasting adaptive immune response.

#### 2.1.1 Innate immune response

TLRs are engaged in inducing antimicrobial factors, including antimicrobial peptides defensin α and β (20, 66), phospholipase A2 and lysozyme (67). When infection occurs, TLRs can recruit leukocytes rapidly from peripheral blood to the infected sites. TLR signaling directly or indirectly induces surface expression of adhesion molecules, including E-selectin and intracellular adhesion molecule (ICAM1), both in circulating inflammatory cells and vascular endothelial cells, priming for leukocytes rolling against the vascular lumen (68). Besides, TLRs also induce chemokines that attract circulating leukocytes to adhere to the vascular wall (69), and subsequently activate these leukocytes (70), then initiating the frontline anti-infection “battle”. In the “battlefield”, TLRs enhance uptake of pathogens by phagocytes (71), strengthen perforin-mediated killing in natural killer cells (NKs) (20), and induce reactive oxidative species (ROS) together with reactive nitrogen species (RNS), both important antimicrobial molecules, from oxidative burst by promoting inducible nitric oxide synthetase (iNOS) and the NADPH oxidase (68, 70, 72, 73) in phagocytic cells (2, 74–76).

These innate immune functions lack specificity: while killing the invading microbes and infected host cells, they are also capable of causing potential damage to both the adjacent local tissues and systemic distal tissues (77, 78).

#### 2.1.2 Adaptive immune response

TLRs are also essential to regulate adaptive immune responses in the process of infection as well (79). TLRs present in antigen-presenting cells (APCs), especially dendritic cells (DCs). APCs indispensably bridge innate and adaptive immunity. TLRs activation in APCs is significant for (1) the maturation of DCs (80, 81); (2) the upregulation of immune co-stimulatory molecules imperative for T cell activation, such as CD40, CD80, CD86 (36, 79, 82); (3) the processing and presentation of pathogenic antigens (83). For T cells, TLR3 and TLR2 promote T cell survival, proliferation, and activation (36, 81, 84). Different TLRs interact with different immunostimulant in the differentiation into T-helper type 1 (Th1) cells, Th17 cells, CD8^+^ cytotoxic T lymphocytes (CTLs), as well as long-lived memory T cells (2, 20, 81).

TLR-3 can recognize viral dsRNA to promote immune-stimulatory Th1 responses (36), which are characterized by differentiation of CD4^+^ T cells into Th1 cells and secretion of immune-stimulatory cytokines (IL-12, IFN-γ, etc.). TLR-2 can interact with peptidoglycan from bacteria to enhance T cell effector functions (84). Meanwhile microbial induction of TLR block regulatory T cell (Treg) suppressive effect, thus abolishing barriers of antigen-specific T cell responses (79). For B cells, TLRs play a critical role in their activation and maturation. TLR4 is engaged in the cellular program that controls B cell proliferation, survival, differentiation and immunoglobin isotype switching (85).

### 2.2 Restoration: tissue repair and remodeling

TLRs also participate in tissue repair and regeneration after direct physical/chemical injury, pathogen attack or inflammation-caused tissue damage (65), which is representative of TLRs’ role in keeping tissue homeostasis. Tissue repair entails restoring lost cell population, mesenchymal architecture, vasculature, and innervation. TLRs help to promote these processes by multiple mechanisms.

TLRs can maintain the survival of parenchymal cells in the tissue by either directly initiating anti-apoptotic signals to inhibit caspase activation (86) or indirectly induce anti-apoptotic factors such as prostaglandins (PG) by up-regulating cyclooxygenase (COX)-2 to sustain survival of both differentiated epithelial cells and progenitor cells in adjacent tissue (87, 88). TLR-signaling promotes PG production and induces migration of PG-secreting stromal cells to the injured epithelium base, facilitating PG to restore epithelial proliferation and cellular organization (87).

TLRs are also engaged in cell proliferation. TLRs are functionally expressed in mesenchymal stem cells (MSCs), which can differentiate into other cell types and restore the lost cell population after injury (89), and TLR signaling is engaged in MSCs proliferation and differentiation. (90). Besides, TLRs are also found to promote fibroblasts, the critical cell type recruited at the late stage of wound healing and creating tissue scaffolds (91), to proliferate and to maintain their cell cycle (54).

TLRs participate in extracellular matrix (ECM) remodeling. This process that shapes the tissue structure during wound healing, by regulating two crucial enzyme groups: the metalloproteinases (MMPs) and the tissue inhibitors of metalloproteinases (TIMPs) (92–95). MMPs degrade ECM components whereas TIMPs inhibit MMP activities (96).In inflammation, TLR3 in mesothelial cells mediates the upregulation of MMP-9 and TIMP-1 (92). TLR2 and TLR3 in chondrocytes regulate the collagenase induction via MMPs (93). TLR9 and TLR4 also can upregulation MMP-9 to remodel ECM (94, 95).

TLR/MyD88/IRAK-4/TRAF-6 signaling contributes to angiogenesis after injury by inducing pro-angiogenic factor, vascular endothelial growth factor (VEGF) and basic fibroblast growth factor (b-FGF)) in macrophages after sensing the danger signal high mobility group box 1 (HMGB1) released from injured cells (97, 98).

In the nervous system, TLR2 and TLR4 participate axonal regeneration and locomotor recovery after peripheral nerve damage and spinal cord injury (99, 100), playing a role in restoring both structural and functional nervous homeostasis. Besides, TLR2 and TLR4 signals mediate microglial proliferation and expansion in brain injury (101), which is a crucial step in the clearance of myelin debris, leading to the room for axon regrowth.

Accumulating evidence demonstrates the protective role of TLRs in injured tissues in multiple organs. The TLR/MyD88 pathway mediates liver regeneration after partial hepatectomy via IL-6 and TNF-α secreted by Kupffer cells (51, 102); MyD88 maintains the rate of contraction, granulation tissue generation, and the density of vasculatures in skin injury by excision (103); TLR/MyD88 signaling is also indispensably engaged in the epithelium restoration of the gastrointestinal tract after dextran sulfate sodium (DSS)-induced injury (104–107).

### 2.3 Inner balance in both “destructive” and “restorative” responses

TLRs are programmed with inner balance in their functions. As discussed above, TLRs promote immunity to attack pathogens, strengthening proliferation to restore tissue structures. They can also induce immune suppression, such as by inducing immunosuppressive cytokine IL-10, to limit the extent of inflammation and immunopathology (108, 109); meanwhile, they can enhance programmed death in the injured cells by ROS and/or TNF-mediated cell cycle rest and apoptosis (110, 111).

The above evidence shows the versatile potentiality in the function of TLRs, conducting a wide range of physiological activities, both “destructive” and “restorative” on tissues. In physiological conditions, these functions are fine-tuned. In tumors, however, the balanced regulation is disrupted. Tumors are cunning enough to “cheat for” the functions that facilitate their expansion but escape from those that suppresses them.

## 3 Versatile TLRs in different aspects of tumor development

**Figure 2:**
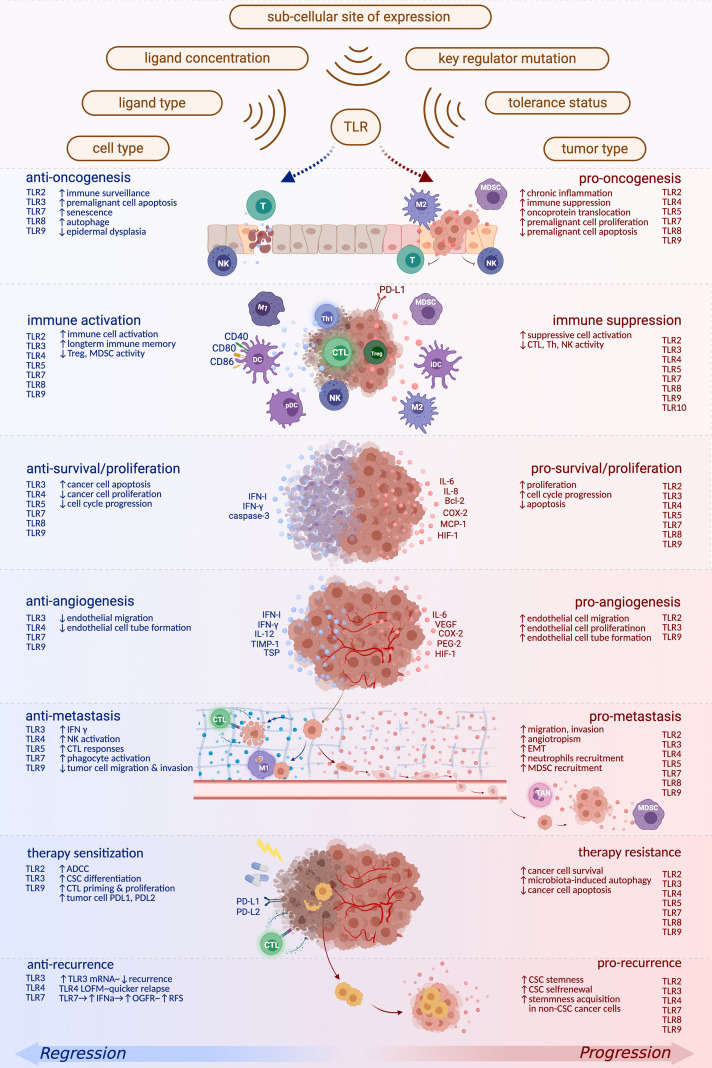
Versatile roles of TLRs in the tumor Many factors have an impact on TLR functions, including but not limited to the type of TLR, the type of cell where the TLR is expressed, the ligand type, the ligand concentration, the sub-cellular site of TLR expression (especially in pathologic conditions), the mutation of key regulators, TLR tolerance status and the type of tumor. TLRs take part in a wide variety of tumor development processes and influence treatment responses. In different conditions, TLRs can exert varied functions, some of which can even be contradictory. TLRs are actively interacting with factors in the TME, thus making TLR function in a dynamic status. The overall effect of TLRs on a tumor is not the result of any single function induced by them; It is the result of the additive, synergistic, antagonistic, or abridged interactions among all the effects they induce. (This figure is created with BioRender.com).Note: TLRs appearing in either pro-tumor or anti-tumor side of this figure represent that there is evidence showing the association or implication of the particular TLR in the respective tumor development stage in a particular situation, and this does not mean the association or implication is universal; the interpretation of TLR functions in tumors should be contextualized.

Beyond their well-established roles in immune activation and tissue repair, accumulating evidence indicates that TLRs exert complex and multifaceted functions during tumor progression. Here we review the roles of each TLR plays in various aspects of tumor development, the regulation of TME, treatment responses and recurrence (**Figure 2**). And we list the role of TLRs in TME on **Table 2**.

**Table 2:** The regulation of TLRs in TME.

The roles of TLRs in TME		References
TLR2 and its heterodimers		
anti-tumor	↑ Th1 immunity	(29)
	↑ CTLs cytolytic activity	(84)
	↓ Treg function	(112, 113)
	↑ M1-like macrophages	(114)
pro-tumor	↑ suppressive function of MDSCs	(115, 116)
	↑ DC polarization to immunosuppressive subtype	(117)
TLR3		
anti-tumor	↑ intra-tumoral CTL recruitment and T cell proliferation	(118–121)
	↑ NKs activation and DC maturation	(118, 120, 122, 123)
	↑ cross-priming of CD8+T cells and T cell responses	(118, 124, 125)
	↓ M2 TAM and MDSCs	(122, 125, 126)
pro-tumor	↑ immunosuppressive molecules (IL-1β, miR-21	(127, 128)
	↑ PD-L1 expression on tumor cells	(129)
TLR4		
anti-tumor	↑ the immunity of DC, CTLs and NKs	(130–132)
	↑ M1 TAM polarization	(133, 134)
	↑ CD8+T cell infiltration	(134)
pro-tumor	↑ MDSCs and M2 TAM polarization	(115, 135, 136)
	↓ T cell proliferation, the activity of CTLs and NK	(137, 138)
TLR5		
anti-tumor	↑ recruitment of neutrophils, NK cells, macrophage, CD11c +cells, and T cells	(139–144)
	↑ M1 polarization, DC activation, CD4+ and CD8+ T cell stimulation	(139, 145–147)
pro-tumor	↑ recruitment of MDSC	(148)
TLR7		
anti-tumor	↑ recruitment of DC, NK and CTL	(149–151)
	↓ Treg population and activity	(151, 152)
pro-tumor	↑ PD-L1 expression	(115, 153)
TLR8		
anti-tumor	↑ pro-inflammatory cytokines by monocytes and myeloid DC	(154)
	↑ IFN-γ by NK	(154)
	↓ T cell senescence	(155–157)
	↓ Treg suppressive effect	(158–160)
	↑ M1 TAM polarization	(161)
pro-tumor	↑ Indoleamine 2,3-dioxygenase (IDO) in monocyte	(162)
TLR9		
anti-tumor	↑ DC accumulation, expansion and maturation	(163–166)
	↑ M1 TAM polarization	(167)
	↑ intratumoral infiltration of CD8+T cell	(163, 167, 168)
	↑ immunity of CD4+ T cell	(163)
	↓ PD-1 expression	(169)
	↑ immunity of B cell	(163, 166)
pro-tumor	↑ M2 TAM polarization	(170)
	↑ PD-L1 expression	(171)

TLR, toll-like receptor; Th1, Type 1 helper T cell; CTL, cytotoxic T lymphocyte; MDSC, myeloid-derived suppressor cell; NK, natural killer cell; DC, dendritic cell; TAM, tumor-associated macrophage.

### 3.1 TLR2 and TLR1, TLR6, TLR10

TLR2 is pattern recognition receptor located on the cell plasma membrane and is known to form homodimer or heterodimers with either TLR1, TLR6, and TLR10 (26, 172–174). (**Figure 1**)TLR1/2 detects triacylated lipopeptides derived from Gram-negative bacteria (25, 26), TLR2/6 senses diacylated lipopeptides produced by mycoplasma (26, 27), and TLR2/10 identifies microbial molecules that overlap with those recognized by TLR1 (172). (**Table 1**) After heterodimerization, TLR1/2 and TLR2/6, but not TLR2/10, need to recruit the TIR domain-containing adaptor protein (TIRAP) before activating the MyD88 pathway (16, 21, 58, 172). (**Figure 1**).

#### 3.1.1 Tumorigenesis

There are studies demonstrating contradictory results on the effect of TLR2 in carcinogenesis. On the one hand, TLR2 participates in the induction of senescence and autophagy, which are homeostatic processes acting as defending machinery against infection and carcinogenesis in the immune system; TLR2 also provides critical immune surveillance suppressing abnormal proliferation during tumor development. TLR2 deletion leads to impoverishment of autophagic flux and defects in immune networks, thus increasing the occurrence of hepatocellular carcinoma (HCC) (175).

On the other hand, TLR2 has been found to be the downstream regulator of the oncogene signal transducer and activator of transcription 3 (STAT3), facilitating cell survival and promoting tumorigenesis. In a gastric tumor model with hyperactivated mutation of STAT3, TLR2 expression in epithelium has been up-regulated; TLR2 subsequently up-regulates genes mediating cell cycle and survival, including *BCL2L1*, *CCND2*, *CCND1*, *BIRC3*, *C-Myc*. Deletion of TLR2 signaling either by *TLR2* knockout or TLR2 antibody blocking reduces gastric tumorigenesis in this model (176). Similarly, in another model of DSS-induced colon cancer, deletion of *TLR2* and *Myd88* has suppressed spontaneous tumor development (177).

#### 3.1.2 Tumor cell survival and proliferation

TLR2 potentially participates in the induction of cancer cell apoptosis. One study showed TLR2 activation up-regulated TNF-related apoptosis-inducing ligand (TRAIL), which is capable of inducing tumor cell apoptosis (178). Otherwise, there is limited evidence supporting TLR2 with this function.

On the other hand, though, more evidence shows the protective role of TLR1/2 or TLR2/6 for tumor cells against apoptosis or the promoting effect on their proliferation. These effects are demonstrated *in vitro* or *in vivo* in lymphoma (179), leukemia (180), multiple myeloma (181), lung cancer (182), gastric cancer (176, 181), hepatocellular carcinoma (28), and colon cancer (29). Such pro-tumor effects are induced by TLR ligation with tumor-derived molecules (28, 182), increased expression of anti-apoptotic genes (BCL2A1, BCL2, BIRC3, CFLAR, IER3, TNFAIP3) as well as decreased expression of tumor suppressor genes (PDCD4, TP53INP1) (181), or the induction of pro-growth cytokines IL-6, IL-8, and MCP-1 (181, 182).

#### 3.1.3 Angiogenesis, invasion, and metastasis

TLR2 is an active regulator in tumor angiogenesis. Oxidative stress-induced endogenous ligand, end products of lipid oxidation, ω-(2-carboxyethyl)pyrrole (CEP) is accumulated in highly vascularized tumors. CEP can be recognized by TLR2, which leads to the production of VEGF and endothelial migration (183). TLR2/6 activation promotes proliferation and angiogenic factor secretion from human intestinal microvascular endothelial cells (HIMEC) and human intestinal fibroblasts, meanwhile enhancing angiogenic responses both *in vitro* and *in vivo* (184). Besides, in human gastric adenocarcinoma urease virulence factor released by the *helicobacter pylori* could stimulate TLR2, not TLR4, and induce hypoxia-induced factor-1α (HIF-1α), promoting angiogenesis and to improve blood supply (30).

TLR2 has shown the ability to enhance cancer cell migration and invasion, as well as exacerbate cancer metastasis in multiple cancer models, including lung cancer (185), pancreatic cancer (31), colorectal carcinoma cells (186), gastric cancer (187), melanoma (32). In these models, the pro-metastatic responses are triggered all by endogenous ligands, such as HMGB1 (31, 33), versican-a tumor secreted proteoglycan (185), HSP90α from tumor cell-released autophagosomes (TRAPs) (32), and 25−Hydroxycholesterol (25−HC)--a product synthesized in inflammatory conditions from cholesterol (187). Mechanisms promoting metastasis encompass TLR2-mediated: IL-6 and transforming growth factor β (TGFβ) induction from cancer stem cells (CSCs), which are a critical cancer cell population responsible for tumor initiation, progression, metastases, relapse, and treatment resistance (33, 188), STAT3 and Smad3 activation (32, 33), PI3K/Akt activation (31), epithelial-mesenchymal transition (EMT) (31) and MMPs expression (187).

#### 3.1.4 Regulation of TME

TLR2 suppresses tumors primarily by immune stimulation. TLR2 and its heterodimers are potent immune stimulators, with much evidence indicating their ability to enhance anti-tumor effector cell functions while limiting immune suppression. TLR2 appears to contribute to the polarization of anti-cancer Th1 immunity, as a TLR2 knockout colitis-associated colorectal cancer (CAC) model showed Th1 immunity was suppressed while the pro-tumor Th17 inflammatory response was enhanced, leading to increased tumor number and size (29). Ligation of TLR1/2 heterodimer on CTLs induces tumor-antigen-specific cytolytic activity *in vivo* (84). Apart from up-regulating the activity of CTLs, TLR1/2 ligation also inhibits Treg function, resulting in tumor remission in a mouse model (112). In addition, by raising the level of macrophage expression of Fcγ IV, TLR1/2 heterodimers can induce macrophage-mediated intratumor Treg depletion (113). Activation of TLR2 has promoted HCC regression in a mouse model via curbing the IL-18 mediated myeloid-derived suppressor cell (MDSC) accumulation and liberating CTL activity (189). A recent study demonstrates that TLR2 ligation with an extracted galactan from fungi is capable of converting the tumor-supporting M2-like macrophages to the tumor-suppressing M1-like macrophages (114). The co-receptor of TLR2, TLR10, participates in pro-inflammatory cytokine/chemokine production of human monocytic THP-1 cells after exposure to TLR ligands (190).

Nonetheless, TLR2 has been reported to impede anti-cancer immunity in the TME as well. TLR2 is involved in the induction of MDSCs, as shown in a murine melanoma model where mouse immature myeloid cells (IMC) transformed to MDSC following the up-regulation of programmed death-ligand 1 (PD-L1) triggered by the interaction between tumor cell extracellular vesicle and TLRs (115). The TLR2/MYD88 pathway also activates the suppressive function of MDSCs, via ligation with tumor-derived exosomal HSP72, leading to autocrine IL-6-mediated STAT3 activation (116). TLR2 mediates DC polarization towards immunosuppressive subtype after binding to tumor-derived versican, resulting in DC production of IL-10 and IL-6 (117). Contrary to the effect of TLR10 in THP-1 cells, recently TLR10 has been found to ameliorate pro-inflammatory cytokine production, including IL-1β, IL-12, granulocyte-macrophage colony-stimulating factor (GM-CSF) and IFNγ in DCs, playing an immune-modulatory role (191).

#### 3.1.5 Treatment response, recurrence and prognosis

TLR2 has been reported to participate in tumor recurrence by influencing CSCs. One group showed TLR2 ligand Pam3CSK4 promoted CSC differentiation and reduced CSC markers in glioblastoma cell lines, indicating the potential of TLR2 signaling in reducing tumor relapse in glioblastoma (34). More studies, however, provided opposite results in other tumor types. In pancreatic, breast, and epithelial ovarian cancer models, TLR2 is found to enrich and enhance the stemness of CSCs, as well as induce their self-renewal (33, 188, 192, 193). This is achieved by paracrine (192, 193) or autocrine (33) HMGB1-TLR2 interaction, involving downstream MyD88-NFκB (188), Hippo-YAP (192), and Wnt/β-catenin (193) pathways.

High expression of TLR2 has been reported to be associated with poor prognosis in several types of cancers. In gastric cancer, Tye et al. (176) have found oncogene STAT3 drives TLR2 up-regulation in gastric cancer tissue; West et al. (181) showed TLR2 expression is higher in tumor tissue than in non-tumor tissue; either high combined STAT3-TLR2 status or high TLR2 expression alone is associated with significantly impaired gastric patient survival (176, 181, 194). In breast cancer, up-regulated TLR2 is associated both with significantly poorer overall survival (OS) and disease-free survival (DFS) (195, 196). In colorectal cancer, TLR2 high expression in tumor tissues is also associated with significantly decreased OS (197).

In lung cancer and pancreatic cancer, however, opposite results have been found. Patients with either increased TLR1 or TLR2 expression in non-small-cell lung cancer (NSCLC) tissue had a significantly longer OS or progression-free survival (PFS) (198). Also, patients of early-stage (tumor size<3cm) pancreatic cancer who had stronger TLR2 expression had better survival (199).

The above evidence indicates that the prognosis implication of TLR2 in cancer varies by cancer type and stage, corresponding with the dynamic and contextualized nature of TLR function.

### 3.2 TLR3

TLR3 primarily exists on endosomes but is also found on the cell membrane of fibroblasts, epithelial cells, and macrophages (13, 14). After binding with double-stranded RNAs (dsRNAs) (25, 35), TLR3 forms into homodimers (25), subsequently activate TRIF pathway (17, 200). (**Figure 1**).

#### 3.2.1 Tumorigenesis

TLR3 has been reported to prevent tumorigenesis, at least partially, by promoting apoptosis in transforming cells and enhancing immune surveillance. Bonnin et al. (201) found in human HCC samples that TLR3 expression was decreased; by using a transgenic mouse model of HCC, they discovered that TLR3 absence leads to accelerated hepatocarcinogenesis; mechanistically, the authors found TLR3 can directly trigger apoptosis in human HCC cells and suppress hepatocyte transformation. Their study suggested the protective role of TLR3 in HCC carcinogenesis. A similar role of TLR3 was shown in a spontaneous orthotopic prostate cancer model, the TRAMP C57Bl6 x FvB F1 Tg(+/-) transgenic mouse model as well, where TLR3 stimulation lead to reduced tumor occurrence, accompanied by increased T cell and NK cell infiltration (202).

#### 3.2.2 Tumor cell survival and proliferation

TLR3 has been reported to play an anti-proliferative and pro-apoptotic role in multiple cancer cell lines. Fan et al. (203) reported that via down-regulation of EGFR/PI3K/AKT signaling, stable expression of TLR3 in breast cancer cell lines suppressed cell proliferation; the TLR3-mediated tumor growth inhibition effect was also observed in a mouse model. In addition, TLR3 activation by poly I:C also induces apoptosis in cells derived from HCC (204), prostate cancer (205), metastatic pharynx carcinoma (206), and human head and neck squamous cell carcinoma (HNSCC) (207). The mechanisms by which TLR3 activation induces apoptosis include: the stimulation of IRF-3 signaling (which plays a key role in both intrinsic and extrinsic apoptotic pathways) (205), the down-regulation of anti-apoptotic proteins (such as survivin) (204, 207), and the augmentation of tumor necrosis factor-related apoptosis-inducing ligand (TRAIL)-induced apoptosis (204).

#### 3.2.3 Angiogenesis, invasion, and metastasis

TLR3 has shown both anti-angiogenic and pro-angiogenic capability in cancer studies. Guo et al. (208) conducted experiments of *in vitro* rat aortic ring outgrowth and tube formation of human umbilical vein endothelial cells (HUVECs) to find that TLR3 activation leads to suppressed angiogenesis. Similarly, Bergé et al. (209) also found treatment with TLR3 agonists poly I:C or small interference RNA (siRNA) inhibited HUVECs from tubular network formation; in the *in vivo* study with a transgenic HCC model, the authors reported TLR3 activation resulted in reduced hepatic arterial vascularization and blood flow. Later, Yuan et al. (210) further discovered in HCC patient tumor samples that TLR3 expression was negatively correlated with the expression of angiogenic cell markers. The above evidence indicates an anti-angiogenesis role of TLR3 in HCC.

However, the pro-angiogenic potential of TLR3 has been shown in *in vitro* studies (211, 212). Paone and colleagues (211) found TLR3 stimulation in the aggressive prostate cancer cell line PC3 induced VEGF production via increasing HIF-1 accumulation; the VEGF containing PC3 culture supernatant induced by TLR3 activation then promoted capillary formation by HUVECs. In another study focusing on angiogenesis mechanism in Epstein-Barr virus (EBV)-related nasopharyngeal carcinoma (212), EBV-encoded RNAs (EBERs) was found to be secreted by cancer cells via extracellular vesicles, which later can infect normal epithelial cells and activate TLR3 as well as RIG-1, resulting in enhanced migratory and tube formation ability of endothelial cells.

TLR3 signaling has shown divergent effects on metastatic potential in different tumors. In neuroblastoma, TLR3 activation seems to prevent metastasis by two mechanisms: 1)direct suppression on the tumor cell activity and 2)activation of the microglia cells, both suppressing the migration and invasion of tumor cells (126, 213). TLR3 expression also acts as a protective role in the process of melanoma metastasis via the induction of IFNγ and NK cell activation, which has been implicated in a B16 melanoma lung metastasis mouse model (214). In breast cancer patients, those with alleles preserving normal function of TLR3 in host peripheral blood lymphocytes have increased metastasis-free survival, compared to those bearing loss-of-function alleles in TLR3 (215), which may suggest the function of TLR3 in preserving host immune surveillance.

On the contrary, there is also evidence supporting the pro-metastasis role of TLR3. In breast cancer patients, the expression of TLR3 in cancer cells instead of peripheral lymphocytes is associated with a higher risk of metastasis (216). Similarly, TLR3 activation in tumor cell lines of lung cancer (217), HCC (218), intestinal epithelial cancer (219), and head and neck cancer (220) induced enhanced cell migratory and invasive ability; such enhancement was associated with TLR3-mediated activation of TANK-binding kinase 1 (TBK1) (218) and secretion of cytokines and chemokines, including IL-6, CCL2, CCL5, CCL20, VEGFA, MMP2, and CXCL10 (217, 219, 220). In addition to cancer cells, TLR3 in normal epithelial cells also takes part in cancer metastasis. Liu et al. (221) discovered a pro-metastatic mechanism: first, primary tumor cells secrete exosomes containing rich small nuclear RNAs; then, these RNAs activate lung epithelial cells through TLR3, subsequently inducing chemokines to recruit neutrophils, which are an important component for the lung pre-metastatic niche. The authors also proved that TLR3-deficient mice developed decreased lung metastases in spontaneous metastatic mouse models (221).

#### 3.2.4 Regulation of TME

TLR3 activation has long been known to directly or indirectly stimulate both innate and adaptive immunity. Firstly, TLR3 activation induces potent type I IFN production (118), Th1-type cytokine production (119), and T cell chemoattractants, such as CXCL10, CCL5, and CXCR3 ligands (120, 121), leading to intra-tumoral CTL recruitment and T cell proliferation (120). Then, TLR3 stimulation also induce NK cell activation (118) and DC maturation by up-regulating surface markers of CD80, CD86, CD40, MHC-II (120, 122, 123). In terms of adaptive immunity, TLR3 activation increases cross-priming of CD8^+^ T cells (118, 124) and enhancement of local T cell responses (125). TLR3 stimulation can inhibit the immunosuppressive function of TAMs by reverting M2 TAM to M1 phenotype (122, 126), and MDSCs, by decreasing their frequencies both locally and systematically (125).

TLR3 also has immunosuppressive potential via inducing cytokines (like IL-1β) or microRNA release. In the TME, in response to the variety of stimuli and signals, different cells can release cytosolic content-containing vesicles into the extracellular space (127). Chen et al. (127) have found such vesicles derived from lung cancer, lung cancer microparticles (L-MP), enriched with non-coding RNAs, can activate TLR3 in macrophages, leading to the release of IL-1β, which subsequently promotes cancer development. MiR-21 is another immunosuppressive molecule that can be induced by TLR3 activation (128). Miao et al. (128) discovered TLR3 agonism in nasopharyngeal cancer cells increased miR-21 secretion, which later induced IL-10 expression in B cells, thereby suppressing CTL activities. In addition, Boes and Meyer-Wentrup (129) showed in their study that while TLR3 stimulation up-regulated MHC I molecules in neuroblastoma cells, it also induced PD-L1 expression on them, indicating the co-existent mechanisms mediated by TLR3 of immune promotion and immune escape in cancer cells.

#### 3.2.5 Treatment response, recurrence and prognosis

TLR3 has been found to take a positive part in sensitizing cancer cells to chemotherapy, radiotherapy, and other biotherapies. TLR3 activation is reported to enhance chemosensitivity in TLR3 positive cancers by 1) down-regulating the drug transporters P-gp and MRP-1 in the malignant cells, leading to an elevated cytoplasmic concentration of the chemotherapeutic agent (222) and 2) inducing autocrine IFN I from tumor cells, which makes them more fragile to DNA toxic agents (223). TLR3 stimulation with poly(I:C) promotes radiosensitivity of cancers as well. Mechanistically, *in vivo* study revealed that poly(I:C) activated TAMs to secrete TNFα, which sensitizes tumor cells to subsequent radiation (37); besides, an *in vitro* study showed in combination with cisplatin, poly(I:C) can suppress c-IAP2 and survivin expression, promote apoptosis and induce G2/M cell cycle arrest in cancer cell lines, resulting in improved radiation attack on these cells (224).

In terms of immune checkpoint blockade (ICB), TLR3-targeted adjuvant ARNAX in tumor vaccine potentiated PD-L1 blockade treatment in an anti-PD-1 resistant lymphoma mouse model, probably through the induction of CTL priming and proliferation (225). In addition, poly (I:C) was found to up-regulate PD-L1 and PD-L2 on glioblastoma cells, facilitating treatment of PD-L1 blockade (226). The anti-tumor effect of another agent, retinoic acid (RA), used for the treatment of acute myeloid leukemia (AML) (227), can also be enhanced by TLR3 activation via up-regulation of RA receptor β (RARβ) in cancer cells, making them more sensitive to RA-mediated apoptosis (23).

However, in a TLR3 high-expression oral squamous cell carcinoma cell line OC2, TLR3 inhibited cytotoxicity of cisplatin (228), suggesting the potential of TLR3 in inducing chemoresistance in some types of cancers.

TLR3 expression and activation have been reported to associate with tumor recurrence. Lower TLR3 mRNA and protein levels in tumor tissues were predictive of shortened recurrence-free survival in HCC patients (201). Lower TLR3 expression in the primary tumor samples determined by real-time PCR (RT-PCR) was associated with biochemical recurrence in prostate cancer patients (229). The evidence here indicates a protective role of TLR3 against cancer relapse.

However, there are discrepant results from other studies. Interestingly, the increased TLR3 expression level in the primary prostate cancer tissues determined by immunohistochemistry(IHC), contrary to the above result, was associated with a higher probability of cancer relapse (230). The contradictory results may indicate discrepant expression levels between TLR3 mRNA and protein; they may also imply a certain level of expression needed for TLR3-mediated protective effect, which should be high enough to maintain TLR3-mediated anti-cancer effects, such as promoting apoptosis, but not too high to initiate pro-cancer effects, such as enhancing proliferation. In breast cancer *in vivo* and *in vitro* studies, TLR3 activation elicited phenotypes of cancer stem cells in breast cancer cells (231), endowing recurrence potential in these cells. Consistent with this finding, in human samples of breast cancer with recurrence, the mRNA level of TLR3 is significantly increased compared to tumor samples without recurrence (216).

TLR3 expression is reported to have a prognostic value in several cancer types. Two separate studies on HCC showed patients with tumor samples that had higher TLR3 expression had longer survival (210, 232). In neuroblastoma, patients with TLR3 positive tumors also had superior 5-year survival compared to those with TLR3 negative tumors (213). In breast cancer, patients carrying loss-of-function TLR3 alleles had a reduced OS after anthracycline-based neoadjuvant chemotherapy (215), which is consistent with the fact that the sensitivity of cancer cells to genome toxic agents relies at least partially on TLR3-TICAM1 signaling (223).

On the contrary, in gastric cancer, TLR3 expression by tumor cells has been found to associate with shortened OS, indicating TLR3 as an aggressive marker in this type of cancer (233).

### 3.3 TLR4

TLR4 locates on the cell surface (4). It’s classic ligand is lipopolysaccharide (LPS) from Gram negative bacteria (25, 38) and fungal mannanes (27). After homo-dimerization (25), TLR4 recruits TIRAP to initiate MyD88 signaling, and it can also recruit TRAM to start TRIF signaling (16, 17, 234). TLR4 is unique among TLRs in its ability to trigger both signaling pathways.

#### 3.3.1 Tumorigenesis

TLR4 seems to play a crucial role in tumor development of many inflammation-associated cancer models, as protection against tumor growth has been observed in TLR4-deficient animals. However, there is a lack of evidence supporting TLR4 to have any influence over tumor initiation.

In a colitis-associated cancer model, TLR4 has been found to facilitate tumor emergence by induction of COX-2 and PGE-2 and by supporting epidermal growth factor receptor (EGFR) signaling (235). In a toxin-induced liver injury-associated carcinogenesis model, both *TLR4* wild type (*TLR4^wt^*) mice, and *TLR4* loss-of-function mutated (*TLR4^mut^*) mice showed similar tumor incidence, while *TLR4^wt^* mice had a significantly larger tumor size and number. STLR4-associated tumor growth enhancement has been associated with tissue repair and regeneration gene expression and the induction of hepatomitogen epiregulin, which facilitates hepatocarcinogenesis as well as inhibiting apoptosis (236). More recently, a group found that hepatic progenitor cell (HPC)-derived myofibroblasts expressed functional TLR4, which can secrete IL-6 and TNFα upon TLR4 activation. These cytokines then assisted HPCs to proliferate and to transform malignantly; meanwhile, the two cytokines are clinically associated with hepatic fibrosis, the predisposed condition of HCC (237).

#### 3.3.2 Tumor cell survival and proliferation

Accumulating evidence has demonstrated that TLR4 signaling maintains or promotes multiple types of tumor cell viability, including colon cancer, human HNSCC, prostate cancer, breast cancer, melanoma, and multiple myeloma (137, 238–242). The activation of TLR4 in tumor cells promoted proliferation and cell-cycle progression and enhanced resistance to apoptosis (137, 240, 242); the silencing of TLR4, however, led to dramatically decreased cancer cell viability (241). Mechanistically, TLR4 exerts the above function partially through AKT/mTOR pathway (238) and NF-κB/IL-6 signaling (239).

However, notably, one study showed that TLR4 could present differential regulation of cancer cell growth depending on the presence status of *TP53*, a tumor suppressor (243). In *TP53^wt^* cancer cells, TLR4 activation inhibited cell growth via induction of IFN-γ, whereas in *TP53^mut^* tumor cells, TLR4 lead to enhanced proliferation due to pro-growth cytokine secretion.

#### 3.3.3 Angiogenesis, invasion, and metastasis

*In vivo* and *in vitro* evidence of the role TLR4 plays in angiogenesis showed contradictory results. *In vivo* study with HNSCC cells demonstrated TLR4 activation promoted VEGF production, suggesting the potential of TLR4 signaling in angiogenesis induction (137). Nevertheless, *in vivo* study with a sarcoma model found that TLR4 stimulation with cationic polymers suppressed tumor angiogenesis, whereas TLR4 deletion led to the disappearance of such suppression (133).

Most studies have defined a pro-tumor role of TLR4 in malignant invasion and metastasis. Both cell lines and patient samples of several types of tumors showed high expression of TLR4 is correlated with tumor metastasis (242, 244, 245). And the activation of TLR4 in tumor cells also promotes invasion and migration (242, 246), whereas inhibition of TLR4 signaling suppresses invasion, migration and mesenchymal transition (247). Nox1 redox signaling (248), sphingosine kinase 1 signaling (249), and AKT/mTOR pathway (238) have been reported to take part in TLR4-mediated tumor cell adhesion, migration, and invasion. In the development of metastasis, EMT and angiotropism, a phenomenon where tumor cells spread along abluminal blood vessel surfaces, are involved. TLR4 was reported to be involved in M2-mediated EMT in pancreatic cancer cells in the way that it significantly up-regulated mesenchymal markers vimentin and snail while down-regulated E-cadherin in cancer cells in the M2-cancer cell coculture system (250). Additionally, TLR4 was also found to recruit and activate neutrophils at the tumor site, which subsequently led to angiotropism in a melanoma model (251). Intratumoral bacteria have been reported to have an impact on tumor progression. Liu et al. (252) have discovered that in cervical cancer Gram^-^ bacteria promoted lymph node metastasis via TLR4/MAPK pathway. More recently, Mattavelli et al. (253) discovered that in triple-negative breast cancer, TLR4 mediated the recruitment of PD-L1^hi^ monocyte in tumor-draining lymph node, which suppressed T cell activity, thus facilitating immune evasion and metastasis.

Interestingly, a breast cancer model presented a divergent impact of the TLR4 deletion in the animal model and tumor cells on cancer metastasis. In the TLR4 knockout (TLR4^-/-^) mice, lung metastatic nodules significantly increased compared to TLR4^wt^ mice, while mice challenged with TLR4^-/-^ 4T1 tumor cells developed fewer metastatic nodules than those challenged with TLR4^wt^ cells (254).

#### 3.3.4 Regulation of TME

The anti-tumor effects of TLR4 depend largely on its immune activation ability. TLR4 has shown to be capable of inducing DC maturation (130), enhancing antigen processing and cross-presentation (131), eliciting TAM M1 polarization (133, 134), elevating levels of IFN and pro-inflammatory cytokines (255–257), promoting CD8^+^ T cell infiltration (134), increasing the number and cytotoxic activity of antigen-specific CTLs and NK cells (131, 132).

However, with the variation in ligands, diseases, types of TLR4-expressing cells, and the actual microenvironmental context, the immune regulating effects of TLR4 may present a spectrum of phenotypes. Therefore, there are also cases when TLR4 facilitates immune suppression and cancer immune escape. TLR4 can elicit immune suppression by induction and expansion of MDSCs (115, 135), and via mTOR-mediated TAM M2 polarization (136). Tumor cells can adopt TLR4 inflammatory and tissue repair signaling, somewhat gaining leukocyte traits by producing multiple cytokines, including IL-6, IL-8 iNOS, IL-12, PD-L1, costimulatory CD28 ligand B7-H2, TGF-β, VEGF, and GM-CSF (137, 138, 258); these cytokines then become the disguise, shielding tumor cells from cytolytic attack by inhibiting T cell proliferation and activity of CTLs as well as NK cells (137, 138).

#### 3.3.5 Treatment response, recurrence and prognosis

TLR4 signaling has been reported to mediate tumor cell resistance to chemotherapeutic drugs in colorectal cancer, ovarian cancer, and prostate cancer (242, 259, 260). In the related studies, down-regulation of TLR4 signaling via TLR4 gene knockdown or siRNA in cancer cells restored chemotherapy induced-apoptosis (242, 259). Mechanistically, PI3K/Akt pathway-elicited anti-apoptosis and microbiota-induced autophagy are involved in TLR4-mediated chemoresistance (242, 260). TLR4 also elicits radioresistance in glioblastoma after activated by the endogenous ligand HMGB1 via the AKT downstream signaling (261).

Many studies revealed an association between TLR4 function and tumor recurrence. High TLR4 expression level is strongly associated with recurrence and poor survival in HCC patients (245); higher TLR4 expression has also been found in relapsed HCC patient samples than in that of non-relapsed (262). In an HCC relapse model, significantly higher tumor burden was seen in the TLR4 response enhanced group than the defective group (263). Cancer stem cells (CSCs) are regarded as the “beating heart of the tumor”, which play a pivotal role in cancer relapse (264). TLR4 is found capable of maintaining cancer stemness. When activated by either exogenous LPS (262) or endogenous HMGB1 (265) released by autophagic cancer-associated fibroblasts (CAFs), TLR4 in tumor cells enhances the stemness features. TLR4-AKT pathway has been reported to involve in this process via increasing CSC population and up-regulating the gene of stemness marker (262).

However, the situation reversed in data from breast cancer patients. TLR4 loss-of-function mutation leads to quicker relapse than normal genotype in patients who had received radiotherapy and chemotherapy (131).

High TLR4 expression has been found to correlate with poor clinical outcomes (including large tumor size, advanced pathologic grade, metastasis, recurrence, advanced clinical staging, shortened DFS and OS) of patients with pancreatic ductal adenocarcinoma (266), breast cancer (267), HCC (245), and colorectal cancer (244, 268).

Curiously, when analyzing the association between cell-type-specific TLR4 expression and clinical prognosis in colorectal cancer patients, Vizoso et al. (269) found expression of TLR4 by tumor stromal fibroblasts was independently associated with a greater probability of relapse and shortened OS, while tumor cell TLR4 expression was associated with a lower rate of relapse.

### 3.4 TLR5

TLR5 exists on the cell surface membrane (4), ligating with bacterial flagellin (35). On activation, TLR5 dimerized to initiate MyD88 pathway (16, 21, 58, 172). (**Figure 1**).

#### 3.4.1 Tumorigenesis

TLR5 has been reported to involve in wound-induced skin cancer formation. In a mouse model with wounded skin, injection of flagellin induced tumor via TLR5, whereas ablation of TLR5 in leukocytes elicited protection against tumor development. The study found that TLR5 signaling in wound site leukocytes up-regulated HMGB1, which had been shown to be associated with chronic skin damage in humans (40).

#### 3.4.2 Tumor cell survival and proliferation

TLR5 has been found to inhibit cancer cell growth upon recognition of flagellin, including breast cancer and non-small cell lung cancer (NSCLC) (270, 271). Zhang and colleagues have demonstrated TLR5 signaling in cancer cells exerts anti-proliferative ability by two mechanisms (1): direct inhibition of cell proliferation and neoplastic-characterized anchorage-independent growth (2), additional inhibition by autocrine molecules secreting from those cancer cells (270). Another study using healthy Balb/C mice presented the pro-apoptotic potential of TLR5 signaling in that systemic injection of flagellin induced cleavage of caspase-3 and its substrate Poly(ADP-ribose) polymerase (PARP) in intestine cells (272).

Nonetheless, in squamous cell carcinoma of tongue (SCCT), TLR5 has been found to promote apoptosis resistance after ligation with extracellular DAMP HSP27 via the NF-κB pathway (39).

#### 3.4.3 Tumor invasion and metastasis

In therapeutic research with models of prostate, colon, mammary cancer, melanoma, and NSCLC, TLR5 showed anti-invasion and anti-metastasis features (139, 140, 271, 273, 274). In these studies, flagellin-based agents are applied either alone or in combination with other treatments; *in vitro*, TLR5 activation can inhibit cell migration and invasion (271), while *in vivo* it suppresses tumor development at distal sites from the primary tumor location (139, 140, 273, 274).

Nevertheless, there is also evidence demonstrating the pro-invasive and pro-metastatic role of TLR5 via several mechanisms. In salivary gland adenocarcinoma, TLR5 activation by flagellin can promote tumor migration and invasion via NF-κB/ERK pathway (275). In colon cancer, TLR5 activation up-regulated the expression of mesenchymal markers, promoting epithelial-mesenchymal transition (EMT), meanwhile strengthening cancer cell migratory (276). The potential of TLR5 in enhancing EMT has also been proved in an ovarian cancer cell line, where flagellin-stimulated SKOV3 cells had up-regulated mesenchymal phenotypes and secreted EMT-related cytokines, which was associated with activation of downstream Wiskott-Aldrich syndrome protein verprolin-homologous 3 (WAVE3) (277).

#### 3.4.4 Regulation of TME

TLR5 has shown anti-tumor capability via immune stimulation in prostate cancer, genital cancer, colon cancer, breast cancer, liver cancer, lymphoma, melanoma and thymoma (139–147). In these conditions, TLR5 activation promotes immunity by eliciting immunomodulatory molecules (IFNγ, CXCL9, CXCL10, IL-8, epithelial cell-derived neutrophil-activating peptide-78, macrophage-inflammatory protein α, etc.) (139, 141–143), recruiting massive immune cells (neutrophils, NK cells, macrophage, CD11c^+^ cells, and T cells, etc.) (139–144), activating anti-tumor immune cells (macrophage M1 polarization, macrophage-mediated tumor cell clearance, DC activation, CD4^+^ and CD8^+^ T cell priming and stimulation, etc.) (139, 145–147), and developing long-term immune memory (139), resulting in potently suppressed tumor progression.

However, there has also been a study reporting TLR5 supported tumor progression by immune suppression. In sarcoma and ovarian cancer models, deletion of TLR5 signaling resulted in delayed tumor growth. In the same study, TLR5 was found to induce IL-6, which recruited MDSCs into tumor sites; the MDSCs then promoted intra-tumoral lymphocytes to secrete galectin-1, curbing anti-tumor immunity and stimulating tumor development (148).

#### 3.4.5 Treatment response, recurrence and prognosis

TLR5 has been shown to play a positive role in drug resistance induction, which is associated with NF-κB activation in SCCT, multiple myeloma, colon cancer and ovarican cancer (39, 276, 278, 279). He and colleagues found binding of TLR5 and HSP27 enhanced chemoresistance in SCCT, while knockdown of TLR5 disrupts NF-κB transactivation, leading to inhibited drug resistance induction (39). Ligation of flagellin with TLR5 leads to doxorubicin resistance in multiple myeloma cells for the elevated secretion of IL-6 (278). In colon cancer cells, TLR5-mediated NF-κB activation brought about reduced miR-125b-5p and increased specificity protein 1 (Sp1) as well as CD248, which altogether resulted in drug resistance (276). TLR5 signaling modulates DC differentiation towards PD-L1^+^ myeloid subtypes in ovarian cancer models, thus hampering ICB treatment efficacy (279).

In contrary, in melanoma (280, 281) and colorectal cancer (281) murine models, TLR5 stimulation sensitized the tumor to the ICB therapy. In addition, in a microarray analysis, a group found a positive correlation between TLR5 and oxaliplatin responsiveness (282).

TLR5 has both negative and positive prognostic evidence in human cancer patients. In oropharyngeal squamous cell carcinoma, the strong expression of chymotrypsin-like protease (CTLP) of an opportunistic oral pathogen, Treponema denticola (Td) (Td-CTLP), is associated with poor disease-specific survival; TLR5 has been found to be positively associated with Td-CTLP, indicating its prognostic value of poor clinical outcome (283). In oral tongue squamous cell carcinoma, TLR5 is an independent predictor of cancer mortality and disease recurrence, all with a hazard ratio over 3.5 (284). However, in NSCLC, strong TLR5 expression was significantly associated with improved prognosis (271).

### 3.5 TLR7

As an intracellular endosome receptor (12), TLR7 recognizes single-stranded RNAs (ssRNAs) (25, 35), and forms into homo-dimers upon ligation (25), which then activate MyD88 pathway (16).

#### 3.5.1 Tumorigenesis

There are conflicting implications of TLR7 in tumorigenesis. TLR7 was found to co-express with CD133, a cancer-initiating cell marker, and sustain regeneration of pluripotent cancer cells (285). Meanwhile, activation of TLR7 in a skin cancer-prone mouse model induced attenuation of epidermal dysplasia and T cell infiltration, thus prohibiting cancer occurrence (286).

#### 3.5.2 Tumor cell survival and proliferation

When TLR7 is activated in tumor cells by either tumor cell-secreted ligands or added agonists, cell proliferation is promoted while apoptosis is suppressed, as reported in studies involving esophageal cancer (287), hepatocellular cancer (268, 288), myeloma (43), leukemia (289), liposarcoma (290), and lung cancer (291). The growth-promoting effect is associated with the activation of pro-inflammatory NF-κB pathway (291) and the subsequent IL-6 induction (43, 290).

Conversely, in studies focusing on squamous cell carcinoma and actinic keratosis (a cutaneous cancer *in situ*), activation of TLR7 by its agonist Imiquimod induced apoptosis of cancer cells (292) and is associated with decreased proliferative cells (41). Similarly, TLR7 ligands significantly suppressed human and murine pancreatic cancer cell proliferation by hampering cell cycle progressing and inducing apoptosis (293).

#### 3.5.3 Angiogenesis, invasion, and metastasis

TLR7 has been reported to inhibit angiogenesis in tumor development. This is achieved by TLR7-mediated: (1) up-regulation of anti-angiogenic factors (TIMP-1, thrombospondin (TSP)) (294–296), (2) down-regulation of pro-angiogenic factors (b-FGF, MMP-9, VEGF, angiogenin, IL-8) (294–297), (3) induction of endothelial cell apoptosis and impediment of cell proliferation (295, 296). (4) inhibition of endothelial migration and invasion. These functions are associated with TLR7-dependent induction of IFN, IL-10, and IL-12 (295).

TLR7 has diverse roles in tumor invasion and metastasis. When activated on tumor cells, TLR7 promotes tumor invasion (287), induces EMT, and recruits MDSCs (298), thus facilitating tumor metastasis. In addition, when activated on immune cells in the TME by tumor cell secreting miRNAs, TLR7 can lead to the release of pro-metastatic cytokines, such as TNF-α and IL-6, contributing to tumor dissemination (290, 299).

Meanwhile, with the strong immune-stimulating capacity, TLR7 activation also demonstrates an anti-metastasis effect. By inducing NK cell activity and tumor-specific CD8^+^ T cell responses, a TLR7 agonist inhibited metastatic colonization of tumor cells in the lung (300).

#### 3.5.4 Regulation of TME

TLR7 activates and maintains immune responses during tumor progression, thereby suppressing tumor growth, which has been reported in conditions of squamous cell carcinomas (152, 301), leukemia (302), lymphoma (149), breast cancer (150, 303), colorectal cancer (304), and skin cancers (150, 297, 304). TLR7 promotes pro-inflammatory cytokine and chemokine production, type I IFN expression (150, 297), splenocytes proliferation, dendritic cell maturation (301, 305, 306), DC, NK, and CTL recruitment to the tumor site (149–151), pDC mobility, and cytotoxicity (297); it protects pDC from apoptosis (307), boost mDC cytolysis activity (308), enhances the sensitivity of tumor cells to CTLs (302), increases intratumoral CTL infiltration (309), suppresses Treg population and activity (151, 152), and reactivates TAMs/tumor infiltrated DCs (TIDCs) (303).

However, when activated by tumor secreted ligands, TLR7 can also facilitate immune escape and suppression, as these ligand-TLR7 signals induce PD-L1 expression (115, 153).

#### 3.5.5 Treatment response, recurrence and prognosis

There are several studies indicating a pro-chemoresistance effect of TLR7 signaling. Chatterjee et al. (310) found activation of TLR7 in mouse lung cancer models lead to enhanced chemotherapeutic resistance, and it is also confirmed in human cancer patients that elevated TLR7 expression was strongly associated with neoadjuvant chemotherapy resistance. The TLR7-associated chemoresistance can be accounted for the activation of downstream NF-κB signaling, which elevated the expression of anti-apoptotic factor bcl-2 (291). Conversely, a decreased expression level of TLR7 and its downstream receptors sensitizes cancer cells to cytotoxic chemotherapeutic agents (311).

However, in a clinical trial (NCT01421017) enrolling recurrent breast cancer patients, TLR7 activation elicited both local and systemic antitumor responses when combining radiotherapy (RT) and cyclophosphamide (CTX), whereas the RT/CTX control group showed no objective responses (312).

TLR7 has both pro-relapse and anti-relapse implications in tumors. In an esophageal cancer study, TLR7 has been shown to participate in the acquisition of stemness in non-stem esophageal squamous cell carcinoma (ESCC) cells after being activated by tumor cell-secreted RNA FMR1-AS1, indicating its potential of endowing self-renewal capacity to the tumor cells (287).

In Urosevic et al.’ s study, via IFNα-induction, the TLR7 agonist Imiquimod was found to up-regulate opioid growth factor receptor (OGFR), which was correlated with longer recurrence-free survival in 52 basal cell carcinoma patients (313).

Increased expression of TLR7 has been implicated in the poor prognosis of patients with colorectal cancer (285), NSCLC (310), and adenocarcinoma (298). Nevertheless, in oropharyngeal squamous cell carcinoma, Jouhi et al. (314) reported low TLR7 expression in human papillomavirus (HPV)^+^ patients was associated with poor disease-specific survival, indicating a protective role of TLR7 in this condition. Protective prognostic association of TLR7 was also found in pancreatic cancer patients, as those who underwent upfront surgery and had higher TLR7 expression in tumor tissues had longer postoperative survival (315).

### 3.6 TLR8

Similar to TLR7, TLR8 also resides on endosomes (12), ligating with ssRNA (25, 35) and pairing to homo-dimers (25) that later activate MyD88 pathway (16).

#### 3.6.1 Tumorigenesis

HPV16 infection is strongly attributing to invasive cervical cancer (316). Increased TLR8 expression is associated with HPV16 clearance (317, 318), indicating the protective role of TLR8 in cervical cancer development.

However, in a study where primary colorectal cancer cells were collected and showed co-expression of TLR8 with CD133, a cancer-initiating cell marker. This study suggested that the existence of TLR signaling in cancer-initiating cells may lead to persistent activation of NF-κB that enables these pluripotent cells to maintain self-renewal, giving rise to the tumor development (285). Recently, Haręża et al. (319) found the homozygous recessive genotype (GG) of TLR8 rs3764879 was associated with 5-fold increase in the risk of high-grade serous ovarian carcinoma (HGSOC) and the carrier of allele G (CG or GG) showed higher TLR8 expression in tumor.

#### 3.6.2 Tumor cell survival and proliferation

TLR8 has opposite roles in the regulation of tumor cell proliferation in different types of tumors. In human cell lines of lung cancer, cervical cancer, and pancreatic cancer, activation of TLR8 leads to NF-κB activation, giving rise to increased Bcl-2 and COX-2 expression, resulting in enhanced tumor cell survival and proliferation (291, 320, 321).

On the contrary, through TLR8/MyD88/p38 pathway, TLR8 activation induces differentiation and growth inhibition in human AML cells (320). More recently, in the AML cell lines, TLR8 selective activation is found to induce cell death via caspase-3 (42).

#### 3.6.3 Angiogenesis, invasion, and metastasis

In human cervical cancer samples, a positive correlation between TLR8 and VEGF expression was found; in a human cervical cancer cell line, a TLR8 agonist also elevated the mRNA level of VEGF (322). These data indicate the pro-angiogenesis potential of TLR8.

There is accumulating data supporting the pro-metastatic and pro-invasive role of TLR8 in tumor progression. In TME, there are multiple endogenous ligands that can activate TLR8 on tumor cells as well as on immune cells, including miRNA and lncRNA. Comito et al. found that immunosuppressive T cells up-regulated TLR8 and miR21 expression in a human prostate cancer cell line, and the TLR8/miR21 axis, in turn, enhances the pro-metastatic features of this cell line by promoting EMT and invasiveness (323). Similarly, Fabbri et al. found lung cancer cell-secreted miR-21 and miR-29a were able to bind human TLR8 in immune cells, initiating a pro-metastatic response (299). In addition, TLR8-AS1, a CAF-regulated lncRNA, was found to up-regulate TLR8 expression in ovarian cancer cells and subsequently activate the downstream NF-κB signaling, leading to the expression of EMT-associated proteins (324). *In vitro* study with colorectal cancer cells found TLR8 positively mediated cell invasion (325). In human patient samples, the pro-metastasis indication of TLR8 is also evidenced. Circulating tumor cells (CTCs)may seed new metastatic colonies in the distant part of the body (326), thus becoming an indicator of disease progression. In a group of CTC-positive metastatic breast cancer patients, expression of TLR8 was significantly increased (327). In another analysis of esophageal adenocarcinoma patients, high nuclear expression of TLR8 was found in those with organ metastasis (328).

#### 3.6.4 Regulation of TME

TLR8 activation has been shown to exert multiple immune-activating effects. Activating TLR8 in monocytes and myeloid DCs leads to the production of pro-inflammatory cytokines, such as TNF-α and IL-12, in NK cells results in enhanced IFNγ production and cytotoxic activity (154); it also increases the expression of costimulatory markers in spleen cells in a mouse model of ovarian cancer (329).

T cell senescence contributes to poor T cell proliferative activity, thus compromising immunity, especially specific immunity led by antigen-specific T cells (155). Both tumor cells and Tregs can induce T cell senescence (156). By preventing the induction of cyclic adenosine monophosphate (cAMP) in tumor cells (157), and suppressing Treg activity, TLR8 signaling hampers T cell senescence, thus preserving the level of immune responses.

TLR8 activation can reverse the suppressive effect of several types of immune cells in the TME. TLR8 can inhibit Treg suppressive responses (158–160). Stimulation of TLR8-MyD88-IRAK4 signaling in Treg (160) and TLR8-mediated glucose uptake and glycolysis in Treg (159) can reverse Treg suppression. γδ1T cells in the TME can potently inhibit DC maturation and function, yet TLR8 ligands can reverse this activity both *in vitro* and *in vivo* (330). TLR8 also induces M1 polarization of TAMs and reverses MDSC-mediated suppression on T cell proliferation *in vitro*, which was further validated in a clinical trial (161).

Despite these abundant data demonstrating the immune-stimulating ability of TLR8, there is also evidence indicating the immune suppression potential of TLR8. Indoleamine 2,3-dioxygenase (IDO) is a negative immune response regulator, and Furset et al. found activation of TLR7/8 in human monocytes induces IDO production and that IDO^+^ monocytes inhibited T cell activation (162).

#### 3.6.5 Treatment response and prognosis

TLR8 activation is reported with evidence both in increasing and reducing drug sensitivity of anti-cancer agents. Lu and colleagues found the TLR8 agonist VTX-2337 can augment the effectiveness of the anti-leukemia monoclonal antibody (mAb), anti-CD20 mAb, rituximab by enhancing the antibody-dependent cell-mediated (ADCC) effect through activating NK cells (154). Ruiz-Torres et al. (331) carried a clinical trial (NCT03906526) and discovered that in HNSCC patient, comparing to anti-PD-1 single therapy, TLR8 agonism and anti-PD-1 combined together showed evident up-regulation of innate immune effector genes and increased mature DCs as well as CD8^+^ T cells, suggesting the facilitating role of TLR8 agonism to ICB.

Nevertheless, stimulation of TLR8 in TLR8 overexpressing human pancreatic cell lines led to increased NF-κB and COX-2 expression, giving rise to increased cell viability, proliferation, and decreased chemosensitivity (332).

TLR8 expression is reported to be a negative indicator of patient outcome. High expression of TLR8 is associated with poor prognosis in cohorts of esophageal adenocarcinoma patients (328) and colorectal cancer patients (where TLR8 expression is an independent prognostic factor) (285). In lung carcinoma, TLR8 expression level and the patient OS are negatively associated as well (333).

### 3.7 TLR9

TLR9 is an endosome transmembrane receptor as well, recognizing nonmethylated CpG motifs normally found in bacterial and viral DNA (25, 35). After ligating the homo-dimer TLR9 (25) recruits MyD88, thus activating the downstream molecules (16).

#### 3.7.1 Tumorigenesis

There are studies reporting both the pro-tumorigenesis and anti-tumorigenesis potential of TLR9. On the one hand, TLR9 activation elicits anti-viral immunity, thus having a protective role against tumor occurrence in several onco-virus infection conditions. Persistent infection of hepatitis B virus (HBV), hepatitis C virus (HCV), HPV16, and Epstein-Barr virus (EBV) can precede multiple types of tumors (334, 335). TLR9 activation have been reported to take part in anti-viral immunity of HBV, HCV, and EBV (336–338); besides, there are efficient anti-HPV16^+^ tumor therapies included TLR9 agonism (339, 340).

On the other hand, continuous TLR9 stimulation can lead to chronic inflammation, which promotes tumorigenesis. High expression of TLR9 is linked to premalignant lesion occurrence in the stomach. Varga et al. (341) reported that an *H.pylori* strain with higher cancer risk enhanced TLR9 expression and activation through a pathogenicity island on the *H.pylori* that facilitated translocation of pro-inflammatory and oncoproteins into the premalignant epithelium. Similarly, a recent study found TLR9 expression in human gastric biopsy tissue is significantly associated with H. pylori infection and intestinal metaplasia (342). Another study focusing on pancreatic cancer showed accelerated oncogenesis via TLR9 ligation in pancreatic stellate cells due to the promotion of inflammation and induction of immune suppression in the TME (343). Similarly, in a spontaneous liver cancer mouse model, the ablation of TLR9 suppressed liver inflammation, fibrosis, and cancer development (344).

#### 3.7.2 Tumor cell survival and proliferation

Depending on the ligand type, the cell type, and cancer type, TLR9 activation can either promote or inhibit tumor cell proliferation, survival, and apoptosis.

TLR9 activation by synthetic CpG motif-containing agonists in tumor cells, including neuroblastoma cells, cervical cancer cells, head and neck cancer cells, B cell lymphoma cells, and B-cell chronic lymphocytic leukemia (B-CLL) cells, can lead to suppressed proliferation and enhanced apoptosis (345–348). Mechanistically, TLR9 stimulation in these cells elicits caspase signaling (346) or slows down the cell cycle when entering the S-phase (348). Interestingly, both the pro-proliferation and pro-apoptosis signaling is programmed in the downstream of TLR9 in normal B cells and B malignant cells; however, the pro-proliferation NF-κB signaling is transient in some B malignant cells (B-cell lymphoma cells and B-CLL cells) followed by increased apoptosis, while it is sustained in primary B cells, resulting in a tumor-suppressive effect (345, 347).

Activated by the synthetic agonist or the endogenous ligands in the following cancer cells, TLR9 showed anti-apoptotic and pro-proliferative activity. In a human myeloma cell line, CpG-stimulated TLR9 saved the cells from apoptosis via the induction of autocrine IL-6 (43). Hypoxia can lead to the release of DAMPs, including the endogenous TLR ligand HMGB1 into the cytosol. In hepatocarcinoma, HMGB1 activates TLR9 and promotes tumor cell proliferation via the downstream p38 activation (44, 45). Another endogenous stimulator of TLR9 is the neutrophil extracellular trap (NET), which is expelled by neutrophils, consisting of protein-stubbed DNA to form a web-like snare, originally discovered to have antimicrobial activity (349). Recently, Tohme et al. (350) discovered that NETs could trigger HMGB1 release, thus promoting cancer cell proliferation via TLR9; consistent with this phenomenon, increased NET formation in liver metastatic colorectal cancer patients who underwent curative resection in this study showed over four times of reduction in disease-free survival. Similarly, Nie et al. (351) demonstrated that in diffuse large B-cell lymphoma, NETs also increased tumor cell proliferation via TLR9 activation, and the downstream NFκB, STAT3, p38 pathways, and NETs were associated with poorer outcomes in patients.

Overall, TLR9 can trigger both pro-survival and pro-apoptotic signals, and the actual effect depends highly on cancer cell types. Endogenous TLR9 ligands tend to lead to malignant cell proliferation; thus, a therapeutic strategy targeting the clearance of these ligands may suppress cancer progression while preserving the TLR9 induced anti-tumor immunity.

#### 3.7.3 Angiogenesis, invasion, and metastasis

There is little evidence supporting the anti-angiogenesis role of TLR9. An *in vitro* study showed the TLR9 stimulating oligonucleotide IMO could inhibit human umbilical vein epithelial cells (HUVECs) from forming capillary (352), yet there is a lack of *in vivo* verification of the anti-angiogenesis effect by the single use of IMO.

On the contrary, several studies supported the angiogenesis-promoting effect of TLR9 activation. Chang et al. (353) found H.pylori can stimulate the human gastric cancer cell line to produce COX-2-mediated PGE2 via TLR9/MAPK signaling, which enhanced HUVEC tubal formation. Sorrentino et al. (354) discovered that TLR9 activation by CpG elicits VEGF production from primary fibroblasts and endothelial cells, which was associated with increased tumor lesions in a mouse lung cancer model. Similar results have also been found by Belmont et al. (355) that TLR9 wild-type mice were with higher VEGF concentration and higher microvessel density compared to TLR9 -/- mice in a lung cancer model. Myeloid cell-mediated revascularization is thought to be an important step in tumor recurrence after radiotherapy (356). In the B16 melanoma mouse model, Gao et al. (357) showed that components released from damaged tumor cells following radiation could activate myeloid cell TLR9, leading to the production of MyD88/NFκB induced IL-6 secretion, resulting in JAK/STAT3 activation, and consequently contributing to neovascularization. In the same study, in TLR9 -/- mice, however, epithelial progenitor cell recruitment was impaired, and angiogenesis was suppressed (357).

Therefore, combination therapy of TLR9 activation and COX2/PEG2 or STAT3 inhibition may improve the efficacy of TLR9-targeted treatment.

TLR9 activation or expression has a contrary effect on tumor invasion and metastasis. In metastatic mouse models of melanoma and colorectal cancer, TLR9 stimulation with CpG oligodeoxynucleotide (ODN) leads to decreased metastases (358, 359). In a brain metastatic model of melanoma, prophylactic CpG use reduced brain metastasis by microglia activation characterized by the increase of microglia-tumor cell contact and phagocytosis (358). In mouse melanoma and colon cancer metastatic models, systemic use of CpG resulted in significantly reduced pulmonary colonization of these tumor cells (359). In human patient breast cancer sample analysis, González-Reyes et al. (216) found high fibroblast-like cell expression of TLR9 was associated with low risk of metastasis.

Opposite results, however, were observed in other types of cancer models or patient samples. In the above sections, we mentioned NET-mediated TLR9 activation; such NET-TLR9 interaction can also lead to enhanced migration and invasion of diffuse large B-cell lymphoma cells and colorectal cancer cells *in vitro* or lymph node cancer dissemination *in vivo* (350, 351). TLR9 expression in CLL cells was found to significantly associated with cell migration (360).Similarly, silencing of TLR9 in PC-3 prostate cancer cells resulted in impaired cell migration, indicating the pro-metastasis potential of TLR9 in prostate cancer (361). Eteshola et al. (362)found the invasive behavior was enhanced in triple-negative breast cancer (TNBC) cells *in vitro* via the activation of cancer cell TLR9 by nucleic-acid-containing DAMPs. In patient-derived cervical squamous cell carcinoma sample analysis, elevated TLR9 expression was found to correlate with increased lymph node metastasis (363). In patient prostate cancer samples, high TLR9 expression was significantly correlated with elevated lymph node metastasis (364).

#### 3.7.4 Regulation of TME

TLR9 activation can induce potent immune stimulation. In DCs, activated TLR9 signaling leads to antigen uptake, DC accumulation and expansion in the tumor (163, 164), DC maturation (165), and various cytokine/chemokine production (166). In pDC, TLR9 stimulation elicits type I IFN production (365, 366). TLR9 agonism enhances macrophage and microglia phagocytosis of tumor cells (358, 367), and also induces TAM polarization towards M1 phenotype in the condition of synthetic agonist (167). TLR9 stimulation in NK cells causes NK activation (368, 369). TLR9 ligation by synthetic agonists can promote intratumoral infiltration of CD8^+^ T cells (163, 167, 168), and optimize CD4^+^ T cell activities, including CD4^+^ T cell-mediated proliferation, exhaustion prevention, and differentiation of functional CTLs (163). Also, TLR9 activation contributes to decreased PD-1 expression on antigen-activated CD8^+^ T cells, preserving their anti-tumor potential (169). There are also studies confirming that TLR9 facilitates humoral immunity by promoting B cell differentiation, antibody production, and antibody-dependent cell-mediated cytotoxicity (163, 166).

Despite the above evidence supporting TLR9’s anti-cancer immune-stimulating role, there are also investigations revealing its immunosuppressive potential when expressed and activated in tumor cells. Bao et al. (170) showed that activated TLR9-NFκB signaling in HCC cells led to chemokine (C-C motif) ligand 2 (CCL2) production, which attracted TAMs into HCC tissues and polarized them towards M2 phenotype. In addition, Zhou et al. (171) found TLR9 activation in HCC cells also induced immune escape via PD-L1 induction in HCC cells. In a prostate cancer study, TLR9 upregulation in cancer cells was found to trigger MDSC accumulation and activation.

Hence, we can see the plasticity of TLR9-mediated immune modulation, which is associated with many factors, including cancer type, the cell type that expresses TLR9, the ligands that bind to TLR9, and so forth.

#### 3.7.5 Treatment response, recurrence and prognosis

TLR9 expression and signaling have an impact on different types of cancer treatment. TLR9 activation by synthetic agonists in several types of cancer cells can lead to enhanced cell sensitivity to radio- and chemotherapy. With CpG ODN treatment, the human lung cancer cell line A549, human epidermoid cancer cell line Hep-2, and human glioma cell line U87 become more fragile to radiation (370–372), which is associated with TLR9/NFκB activation and NO production (370). Intratumoral administration of CpG improved radiotherapy response in a murine sarcoma model (373).TLR9 activation also improved the effectiveness of the chemotherapeutic agents, gemcitabine and Alimta, in treating NSCLC, confirmed in NSCLC xenograft models (374). Additionally, a low concentration of CpG ODN C792 can synergize with the non-platinum chemotherapeutic agent, the proteasome inhibitor bortezomib, in enhancing cytotoxicity to multiple myeloma cell lines (375). In murine colorectal cancer model, ODN1585 sensitized ICB treatment via TLR9 activation, which was associated with reduced peritoneal resident macrophages (376).However, data from a refractory melanoma clinical trial (NCT03445533) showed no significant improvement in objective response rate and OS by adding TLR9 agonist Tilsotolimod to ICB treatment (Ipilimumab) (377).

However, studies of the colorectal cancer cell line and the ovarian cancer cell line showed TLR9 stimulation induced chemoresistance (378, 379). Li et al. (379) have found 5-FU and oxaliplatin up-regulated TLR9 expression in colorectal cancer cell lines and subsequently strengthened IRAK4 and NF-κB activity, which leads to treatment resistance. Besides, Cai et al. (378) demonstrated that the TLR9 agonist attenuated the inhibitory effect of cisplatin on TLR9 expressing human ovarian cancer cell line SKOV3, while TLR9 antagonist sensitized chemoresistant SKOV3 cells to cisplatin. CSCs are major contributors to chemoresistance and recurrence. Liu et. al (380)found hypermitophagy in human lung cancer stem cells, which lead to CSC proliferation mediated by lysosomal mtDNA-TLR9-Notch1-AMPK activation.

Seemingly contrary results appear in the above evidence. NF-κB activation can lead to both therapy sensitization (370) or resistance (379). This indicates that not only TLR9 but also its downstream molecules are programmed with pathways leading to differentiated functions, some of which may contradict each other; at the same time, it is a reminder that cancer studies with TLRs should be highly contextualized in specific ways simulating real clinical conditions, taking the tumor type, cell type, tumor stage, etc. into consideration.

TLR9 is reported to have a positive correlation with tumor recurrence. In recurrent tumor samples of prostate cancer and breast cancer, the mRNA expression of TLR9 is significantly increased (216, 230); in prostate cancer, high TLR9 expression is significantly associated with a higher risk of biochemical recurrence (230). In mouse models of melanoma, bladder cancer, and colon cancer, systemic TLR9 inhibition delayed tumor relapse after radiotherapy, which is also associated with myeloid cell JAK/STAT3 activation by TLR9/MyD88/NFκB mediated IL-6 secretion (357).

TLR9 expression has prognostic value in several types of cancers. TLR9 tends to have a protective role in TNBC patients. Low expression of TLR9 in the tumor tissue is associated with significantly decreased disease-specific survival (381); besides, the combinatory prognostic marker of low TLR9 expression and low CD8^+^ T cell frequency in the TNBC tumor tissue is associated with the worst disease-specific survival, while high TLR9 expression and high CD8^+^ T cell frequency is with the best (382). In renal cell carcinoma (RCC) patients, TLR9 is also associated with favorable RCC-specific survival.

However, in other types of cancers, there are reports showing a negative association between TLR9 expression and good prognosis. TLR9 expression in the tumor tissue of prostate cancer and glioma is an independent prognostic indicator for PFS, with strong TLR9 expression associated with shorter PFS (383, 384). In esophageal adenocarcinoma, high TLR9 expression in the tumor is associated with significantly decreased 10-year survival, together with advanced tumor stage, poor differentiation, and enhanced proliferation (385). A recent study with patient tumor sample of diffuse large B-cell lymphoma (DLBCL) showed that high TLR9 expression is associated with poorer outcome, identified by refractory disease or relapse (386).TLR9 expression on immune cells is also correlated with patient survival. Mononuclear cell expression of TLR9 in early-stage NSCLC tissue was found to contribute to worse survival (355).

## 4 Conclusion and perspective

TLRs participate “destruction” (immune attack to pathogens and infected host cells) and “restoration” (tissue repair after damage to restore the original structure) to maintain physiological homeostasis. And in tumors, TLRs exhibit multifaceted roles in tumor development and TME. Many factors influence TLR functions. Here we list possible factors (include but not limited to the following aspects) and examples of how they impact TLR functions. Tumor type: while TLR5-deficiency slows down tumor progression of sarcoma and ovarian cancer in mice, it speeds up tumor progression in breast cancer (387). Cell type: while TLR3 activation induces apoptosis in malignant prostate cancer cells, it hardly elicits this effect in normal prostate epithelial cells (205); similar results are also observed in human primary and metastatic HNSCC cells, with metastatic cells more sensitive to TLR3 activation-induced apoptosis (388). Sub-cellular expression site: in HCC cells, whereas membranous TLR3 activation hardly influences cell viability, cytoplasmic TLR3 stimulation significantly elicits an apoptotic effect (204). Mutation of key regulators affecting TLR pathways: TLR4 activation in normal TP53 cancer cells leads to suppressed cell growth, whereas it improves cancer cell growth when TP53 is mutant (243). Ligand type: in B-cell lymphoma, while exogenous CpG ODN causes cell apoptosis via TLR9 (345), endogenous NETs released by tumor cells enhanced cell proliferation (351). Ligand concentration: while poly (I:C) used at normal concentration inhibits HCC cell growth via TLR3, lower concentration does not show a similar effect but elicits HCC invasion and metastasis (218). Stimulation tolerance: whereas primary activation of TLR7 by imidazoquinoline leads to TNF-α production by mononuclear cells, re-stimulation fails to induce the same effect within five days after the primary attempt (311).

TLRs are at the top of various molecular pathways that lead to a wide range of bioactivities. They are fine regulated by multiple factors in the TME; the effects they elicit, in turn, shape the TME, thus making TLR functions in a dynamic and continuously changing status.

In summary, the overall effect of TLRs on a tumor is not the result of any single function induced by them. It is the result of a tune played by two powers: the power of maintaining homeostasis by the healthy host system and the power of breaking it by tumors. One power plays a “harmonious” tune (regarded as “good” or anti-tumor), while the other plays a “dissonant” tune (regarded as “bad” or pro-tumor). The multifaceted potential of TLR, such as the induction of proliferation, differentiation, apoptosis, chemotaxis, immune activation, and immune tolerance, makes the keyboard that can be utilized by both of the two powers composing their respective tunes. The distance of the final melody created (the actual body status) from harmony(homeostasis) depends on the overall influence left by the battle of “good” and “bad”, which results from the additive, synergistic, antagonistic, or abridged interactions between each note(effect) created by the two powers.

Given the dual and context-dependent roles of TLRs in tumor progression, their overall effect on tumor progression represents a dynamic balance between host homeostatic regulation and tumor-driven disruption. To dissect these dual functions, advanced technologies such as single-cell RNA sequencing, spatial transcriptomics, and multi-omics integration are indispensable. These approaches can resolve the cell type-specific expression and signaling landscapes of TLRs within both tumor and immune compartments, providing critical insights into their context-dependent roles (389, 390).

Furthermore, coupling these findings with precision drug-targeting systems—such as nanoparticle-based or localized delivery—may enable selective modulation of TLR signaling in specific cell populations (391–393).This strategy could amplify beneficial anti-tumor responses while minimizing pro-tumor or immunosuppressive effects, representing a promising direction for next-generation TLR-targeted immunotherapies.
